# Successful High-Dosage Monotherapy of Tigecycline in a Multidrug-Resistant *Klebsiella pneumoniae* Pneumonia–Septicemia Model in Rats

**DOI:** 10.3390/antibiotics9030109

**Published:** 2020-03-03

**Authors:** Hessel Van der Weide, Marian T. Ten Kate, Denise M. C. Vermeulen-de Jongh, Aart Van der Meijden, Rixt A. Wijma, Stefan A. Boers, Mireille Van Westreenen, John P. Hays, Wil H. F. Goessens, Irma A. J. M. Bakker-Woudenberg

**Affiliations:** Department of Medical Microbiology & Infectious Diseases, Erasmus University Medical Center Rotterdam (Erasmus MC), 3015 GD Rotterdam, The Netherlands; hessel.vanderweide@gmail.com (H.V.d.W.); m.tenkate@erasmusmc.nl (M.T.T.K.); d.m.c.dejongh@hotmail.com (D.M.C.V.-d.J.); a.vandermeijden@erasmusmc.nl (A.V.d.M.); r.wijma@erasmusmc.nl (R.A.W.); stefan_boers@me.com (S.A.B.); m.vanwestreenen@erasmusmc.nl (M.V.W.); j.hays@erasmusmc.nl (J.P.H.); i.bakker-woudenberg@erasmusmc.nl (I.A.J.M.B.-W.)

**Keywords:** tigecycline, meropenem, pneumonia, septicemia, *Klebsiella pneumoniae*, antibiotic resistance

## Abstract

**Background:** Recent scientific reports on the use of high dose tigecycline monotherapy as a “drug of last resort” warrant further research into the use of this regimen for the treatment of severe multidrug-resistant, Gram-negative bacterial infections. In the current study, the therapeutic efficacy of tigecycline monotherapy was investigated and compared to meropenem monotherapy in a newly developed rat model of fatal lobar pneumonia–septicemia. **Methods:** A *Klebsiella pneumoniae* producing extended-spectrum β-lactamase (ESBL) and an isogenic variant producing *K. pneumoniae* carbapenemase (KPC) were used in the study. Both strains were tested for their in vitro antibiotic susceptibility and used to induce pneumonia–septicemia in rats, which was characterized using disease progression parameters. Therapy with tigecycline or meropenem was initiated at the moment that rats suffered from progressive infection and was administered 12-hourly over 10 days. The pharmacokinetics of meropenem were determined in infected rats. **Results:** In rats with ESBL pneumonia–septicemia, the minimum dosage of meropenem achieving survival of all rats was 25 mg/kg/day. However, in rats with KPC pneumonia–septicemia, this meropenem dosage was unsuccessful. In contrast, all rats with KPC pneumonia–septicemia were successfully cured by administration of high-dose tigecycline monotherapy of 25 mg/kg/day (i.e., the minimum tigecycline dosage achieving 100% survival of rats with ESBL pneumonia–septicemia in a previous study). **Conclusions:** The current study supports recent literature recommending high-dose tigecycline as a last resort regimen for the treatment of severe multidrug-resistant bacterial infections. The use of ESBL- and KPC-producing *K. pneumoniae* strains in the current rat model of pneumonia–septicemia enables further investigation, helping provide supporting data for follow-up clinical trials in patients suffering from severe multidrug-resistant bacterial respiratory infections.

## 1. Introduction

Recent data from the U.S. indicate that Gram-negative bacteria are responsible for more than 30% of hospital-acquired infections [1]. A complicating factor in the treatment of infections caused by Gram-negative bacteria is the worldwide increase of antimicrobial resistance. In particular, Gram-negative bacteria resistant to carbapenem antibiotics—due to the presence of plasmid-mediated multidrug resistance, such as with extended-spectrum β-lactamase (ESBL) or *Klebsiella pneumoniae* carbapenemase (KPC)—can no longer be effectively treated with β-lactam antibiotics and tend to be susceptible only to “drugs of last resort” [2,3].

Tigecycline (an antibiotic belonging to the class of glycylcyclines) is one such drug of last resort, although resistance to tigecycline has been reported [4]. Further, although the United States Food and Drug Administration (FDA) issued a warning in 2013 relating to an apparent increased death rate during antibiotic treatment using tigecycline (loading dose of 100 mg followed by 50 mg every 12 h) [5], multiple studies have suggested that using a high dose of tigecycline could actually improve the outcome in comparison to conventional dosing [6,7]. Additionally, the European Committee on Antimicrobial Susceptibility Testing (EUCAST) has recently recommended the use of high-dosage tigecycline treatment in seriously ill patients infected with multidrug-resistant bacteria [8]. As well as dosing issues, another debate about the role of tigecycline involves its use in monotherapy as opposed to combination therapy, particularly in the treatment of multidrug-resistant, Gram-negative bacterial infections. This issue is complicated by the fact that comparative clinical efficacy studies into tigecycline are complex, involving differing patient characteristics, various combinations of antibiotics, and differences in the nature and severity of infections [9,10]. 

To investigate this issue further, in rats we developed a model of bilateral pneumonia–septicemia, in which we were able to compare the therapeutic efficacy of individual antibiotics at similar conditions of severity, duration of infection, and host defense. This novel pneumonia–septicemia model was based on a previously established rat model of unilateral pneumonia–septicemia (in which one lung was left uninfected), which was intended for antimicrobial pharmacokinetic and pharmacodynamic studies [11]. Further, in order to mimic the actual clinical situation, the rat infection model is fatal if left untreated, with rat survival (based on rats reaching humane endpoints) as a treatment outcome parameter. In the present study, we established and characterized the lobar pneumonia leading to fatal septicemia, which was caused by either an ESBL-positive *K. pneumoniae* strain or its isogenic KPC-positive variant. The model was used to investigate the therapeutic efficacy of tigecycline as monotherapy as compared to meropenem monotherapy, a potent carbapenem antibiotic that serves as the conventional treatment for multidrug-resistant, Gram-negative bacterial infections [12]. 

## 2. Materials and Methods

### 2.1. Bacterial Strains

*K. pneumoniae* American Type Culture Collection (ATCC) 43816™ (capsular serotype 2) was used as a parent strain to generate the isogenic ESBL-producing variant *K. pneumoniae* ESBL EMC2003 (referred to in this study as *K. pneumoniae* ESBL) and the isogenic KPC-producing variant *K. pneumoniae* KPC EMC2014 (referred to in this study as *K. pneumoniae* KPC) via bacterial conjugation with an ESBL-producing or KPC-producing clinical isolate and subsequent selection on antibiotic agar plates. The stability of both plasmid-containing strains was assessed through five consecutive passages in Mueller–Hinton II broth (Becton Dickinson BV, Vianen, The Netherlands). The virulence of the bacterial strains was maintained by rat lung passage every 12 months. ATCC Quality Control strains *K. pneumoniae* ATCC 13883™ (WT), *K. pneumoniae* ATCC 700603™ (ESBL), and *K. pneumoniae* ATCC BAA1705™ (KPC) were used as reference strains.

### 2.2. Genotypic Characterization of Bacterial Strains

Due to the complex relationship between antibiotic resistance and virulence factors, it is important to report the genotypic data of bacterial strains used in microbiological studies [13,14]. Polymerase chain reaction (PCR) assays were used to verify the presence of the following resistance genes: cefotaxime (CTX)-M β-lactamase groups 1, 2, 8, 9, 25 [15]; temoniera (TEM) β-lactamase [16]; sulfhydryl reagent variable (SHV) β-lactamase [17]; oxacillinase (OXA) 1-like β-lactamase groups 1, 48 [18,19]; KPC [20]; and New Delhi metallo-β-lactamase 1 (NDM) [21]. Multilocus sequence typing (MLST) was used to investigate genetic relatedness. For this, partial DNA sequences of the seven housekeeping genes gapA, infB, mdh, pgi, phoE, rpoB, and tonB were generated and compared using a published high-throughput MLST (HiMLST) strategy [22] that had been adapted for *K. pneumoniae* isolates [23]. To further assess the genetic relation between the strains, pulsed-field gel electrophoresis (PFGE) was used as previously described [24].

### 2.3. Antibiotics and Anesthetics

The antibiotics used were amikacin hydrate, ceftazidime hydrate, ciprofloxacin hydrochloride monohydrate, colistin sulfate salt, gentamicin sulfate, meropenem trihydrate, norfloxacin, tigecycline, tobramycin sulfate salt, trimethoprim, and sulfamethoxazole (Sigma-Aldrich Chemie BV, Zwijndrecht, the Netherlands). For co-trimoxazole, the ratio of trimethoprim/sulfamethoxazole was 1:19. Meropenem was combined with cilastatin sodium (Bosche Scientific LLC, New Brunswick, NJ, USA) (1:1) when used in vivo to inhibit the function of dehydropeptidase enzyme, which is a known catalyst of meropenem in rats [25]. 

The anesthetics used were medetomidine hydrochloride (Eurovet Animal Health BV, Bladel, The Netherlands), fentanyl citrate (Hameln Pharma Plus GmbH, Hameln, Germany), and midazolam hydrochloride (Actavis Group PTC ehf, Hafnarfjörður, Iceland), which were administered together intraperitoneally at 0.5 mg/kg, 50 μg/kg, and 5 mg/kg, respectively. Antagonists were atipamezole hydrochloride (Vetoquinol BV’s, Hertogenbosch, the Netherlands), naloxone hydrochloride dihydrate (Hameln Pharma Plus GmbH), and flumazenil (Fresenius Kabi Nederland BV, Zeist, The Netherlands), which were administered together intraperitoneally at 1.5 mg/kg, 0.7 mg/kg, and 0.3 mg/kg, respectively.

### 2.4. Antimicrobial Susceptibility of K. pneumoniae Strains

The antimicrobial susceptibility of bacterial strains was assessed by determination of the minimum inhibitory concentration (MIC) using the broth microdilution method following EUCAST guidelines [26], which represents the lowest antibiotic concentration needed to inhibit bacterial growth under standardized and predefined laboratory conditions. Phenotypic characterization of bacterial strains was based on antimicrobial susceptibility as determined using the VITEK^®^2 system and AST-N344 Gram-Negative Susceptibility Cards (bioMérieux Benelux BV, Zaltbommel, The Netherlands).

### 2.5. Concentration- and Time-Dependent Bactericidal Activity of Antibiotics In Vitro

The bacterial killing capacity of meropenem and tigecycline was investigated using the time–kill kinetics (TKK) assay, as previously described [27]. Two-fold increasing antibiotic concentrations were used representing 1/16-fold up to 32-fold the epidemiological cutoff value (ECOFF) values of the two antibiotics, as reported by EUCAST [28]. The ECOFF of *K. pneumoniae* for meropenem is 0.125 mg/L and for tigecycline was 1 mg/L, but has recently been increased to 2 mg/L. Samples were centrifuged at 12,500× *g* for 5 min to avoid drug carry-over, serially 10-fold diluted, and subcultured on Mueller Hinton II agar plates (Becton Dickinson BV, Vianen, The Netherlands) for colony-forming unit (CFU) counts after 24 h at 37 °C. At 24 h, changes in antibiotic susceptibility were determined via MIC assay using subsamples of 1 mL to prepare bacterial inocula.

### 2.6. Animals

Specified pathogen-free (SPF) male strain RP–AEur–RijHsd albino rats bred at the Laboratory Animals Center of Erasmus University Medical Center Rotterdam (Erasmus MC) were used. Rats (age, 10–18 weeks; body weight, 250–350 g) were housed individually in ventilated cages and were given food and water ad libitum. Rats were randomly allocated to experimental groups once they reached the appropriate age and body weight. Group sizes were based on estimates of the hazard ratio. Euthanasia was applied by CO_2_ exposure when humane endpoints were reached or at termination of experiments. 

### 2.7. Ethics

Animals were maintained and handled in accordance with the Guidelines for Accommodation and Care of Animals (European Convention for the Protection of Vertebrate Animals Used for Experimental and Other Scientific Purposes). All animal procedures were performed in accordance with the Dutch Animal Experimentation Act (BWBR0003081), which meets the requirements of the European Union Animal Directive (2010/63/EU). Experimental procedures were approved by the Institutional Animal Care and Use Committee of the Erasmus MC. The current study was designed to abide by the “three Rs” principles of animal research (replace, reduce, refine) wherever possible [29] and was written to conform to the animal research: reporting of in vivo experiments (ARRIVE) guidelines for reporting animal research [30].

### 2.8. Rat Model of Pneumonia–Septicemia

Acute bilateral pneumonia–septicemia in rats (referred to in this study as ESBL pneumonia–septicemia or KPC pneumonia–septicemia) was established in groups of 11 rats using suspensions of washed bacteria in the logarithmic phase of growth. After intubation and cannulation of the trachea under anesthesia, rats were held in a vertical position and the lungs were inoculated with 60 µL PBS containing the respective absolute lethal dose (LD^100^) inocula: 2 × 10^6^ *K. pneumoniae* ESBL or 6 × 10^7^ *K. pneumoniae* KPC. An implantable programmable temperature transponder (IPTT)-300 (Plexx BV, Elst, The Netherlands) was implanted subcutaneously. Infected rats were monitored 12-hourly for 11 days to assess the disease progression and pre-defined humane endpoints by changes in body temperature and body weight, as well as by external symptoms of disease, including ungroomed appearance, pallor, nose bleeding, lack of reactivity, inactivity, instability, or abnormal breathing of rats. Rat survival was based on rats reaching humane endpoints, at which point euthanasia was applied by CO_2_ exposure. After dissection, bacteria isolated from lungs and blood were identified by matrix-assisted laser desorption/ionization time-of-flight (MALDI-TOF) mass spectrometry (Bruker Daltonics, Bremen, Germany) to rule out co-infection, a pre-established exclusion criterium. 

### 2.9. Characterization of Pneumonia–Septicemia Model

The early phase of infection was characterized at 24 h and 48 h after initiation of infection by viable counts of *K. pneumoniae* in lungs and blood taken from 6 rats per time point. Blood obtained by cardiac puncture was collected from euthanized rats in lithium heparin tubes (Sarstedt BV, Etten-Leur, The Netherlands). The five lung lobes were collected separately in 2 mL PBS each and homogenized using the gentleMACS™ Octo Dissociator (Milteny Biotec BV, Leiden, The Netherlands). Blood and lung homogenates, either undiluted or in 10-fold dilutions, were subcultured on Mueller–Hinton II agar plates (Sigma-Aldrich Chemie BV, Zwijndrecht, The Netherlands) for CFU counts after 24 h at 37 °C. In blood plasma samples, the alanine aminotransferase (ALAT) and aspartate aminotransferase (ASAT) levels were assessed for hepatic function, and the creatinine and blood urea nitrogen (BUN) levels were assessed for renal function. Histopathological examination of the infected lungs at 24 h after infection was performed in 2 rats with ESBL pneumonia–septicemia and 2 rats with KPC pneumonia–septicemia. In sacrificed rats, in situ lungs were fixated with 10% formalin under constant pressure to re-expand the lungs. Segments of the left lung were dehydrated in ethanol and toluol, and finally embedded in paraffin. Paraffin-embedded tissues were cut into 4-µm sections, from which one in every 15 cuts was used for hematoxylin–eosin (HE) staining and one for Gram staining.

### 2.10. Antimicrobial Treatment of Pneumonia–Septicemia

The therapeutic response to meropenem was determined in rats with ESBL pneumonia–septicemia and rats with KPC pneumonia–septicemia. The response to tigecycline was only investigated in rats with KPC pneumonia–septicemia, as this had already been investigated in a previous unilateral pneumonia–septicemia rat model caused by the identical *K. pneumoniae* ESBL strain [11]. Treatment groups consisted of 11 rats. Antibiotics were always administered intraperitoneally at a volume of 2.5 mL/kg 12-hourly for 10 days, starting at 24 h after initiation of infection. Meropenem in doses ranging from 6.25 to 25 mg/kg/day were administered by 2-fold increases until a minimum effective dose (MED) of meropenem was reached, at which all rats survived. Tigecycline was administered at 25 mg/kg/day (i.e., the MED necessary for survival of all rats with ESBL pneumonia–septicemia from a previous study) [11]. Disease progression was monitored 12-hourly. Rats reaching humane endpoints were euthanized and dissected to rule out co-infection.

### 2.11. Pharmacokinetics of Meropenem

Meropenem plasma concentrations were determined in 3 rats with ESBL pneumonia–septicemia. Rats received 3 doses of 25 mg/kg/day meropenem 12-hourly, starting at 24 h after initiation of infection. Blood obtained by tail vein puncture was collected in ethylenediaminetetraacetic acid (EDTA) tubes (Sigma-Aldrich Chemie BV, Zwijndrecht, The Netherlands). Meropenem concentrations were determined by high-performance liquid chromatography-mass spectrometry (HPLC-MS) [31]. The lower limit of quantification of meropenem was 0.20 mg/L. The calibration line was linear from 0.20 to 40.0 mg/L, with a determination coefficient of at least 0.995. Rat samples were prepared together with calibration standards and quality control samples at three levels. The values of the volume of distribution (V), the clearance (CL), and the concentration half-life time (t_1/2_) of the meropenem elimination process were calculated using a PKSolver 2.0 one-compartment model of extravascular administration with time delay to account for absorption [32]. The V and CL were presented as a fraction of the absorbed dose (bioavailability, F). The protein binding of meropenem in rats has been reported as 22.4% [33]. As only the unbound fraction of the drug is responsible for its antimicrobial effect, the values of (1) the cumulative percentage of a 12-h period that the unbound fraction of drug exceeded the MIC (*ƒ*T > MIC) and (2) the highest unbound meropenem concentration reached in blood plasma (*ƒ*C_max_), were calculated based on unbound meropenem concentrations.

### 2.12. Statistical Analysis

Kaplan–Meier rat survival curves were generated, and statistical differences in rat survival rates were calculated using the log rank test using Prism 5.01 (Graphpad Inc., San Diego, CA, USA).

## 3. Results

### 3.1. Bacterial Strains

The results from HiMLST (Supplementary Table S1) and PFGE assays confirmed the isogenicity of the *K. pneumoniae* ESBL and *K. pneumoniae* KPC strains. ESBL and KPC resistance genes were detected via PCR, as expected in the respective strains (Supplementary Table S1).

### 3.2. Antimicrobial Susceptibility of K. pneumoniae Strains

The antimicrobial susceptibility of *K. pneumoniae* ESBL and *K. pneumoniae* KPC for clinically relevant antibiotics was assessed by MIC determination and compared with the MIC values of 3 ATCC Quality Control reference strains. The *K. pneumoniae* ESBL strain was resistant to ceftazidime and tobramycin, and susceptible to meropenem based on MIC values (Table 1). The isogenic *K. pneumoniae* KPC strain was resistant to ceftazidime, tobramycin, and meropenem. According to the EUCAST 2020 guidelines, both *K. pneumoniae* isolates are resistant to tigecycline, as are all the reference strains used in this study [26]. However, both *K. pneumoniae* isolates still fell within the antimicrobial wild-type distribution of *K. pneumoniae* based on the ECOFF value of tigecycline for *K. pneumoniae* (2 mg/L), and can be considered representative of the tigecycline susceptibility of wild-type *K. pneumoniae* populations—albeit on the high end of the wild-type distribution [28]. 

VITEK^®^2 data (Supplementary Table S2) showed that *K. pneumoniae* ESBL and *K. pneumoniae* KPC were both resistant to a wide range of β-lactam antibiotics and aminoglycoside antibiotics.

### 3.3. Concentration- and Time-Dependent Bactericidal Activity of Antibiotics In Vitro

**Figure 1:**
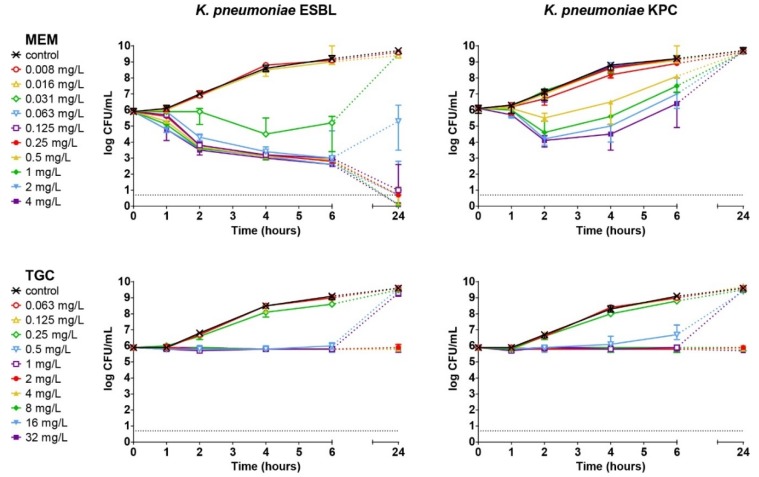
Concentration- and time-dependent bactericidal activity of meropenem (MEM) and tigecycline (TGC) against *K. pneumoniae* strains. Bacterial cultures of *K. pneumoniae* ESBL or *K. pneumoniae* KPC were exposed to two-fold increasing concentrations of antibiotic for 24 h. Shown are the median ± range of in triplicate experiments. The dashed grey line indicates the lower limit of quantification of log 0.7.

The in vitro killing capacity of meropenem and tigecycline against *K. pneumoniae* ESBL or *K. pneumoniae* KPC over 24 h was determined using TKK assays. In the absence of antibiotics, the bacterial populations rapidly increased within 24 h of incubation (Figure 1). Meropenem showed time-dependent killing against *K. pneumoniae* ESBL. A concentration of ≥0.125 mg/L meropenem resulted in a 100-fold reduction in bacterial numbers after 2 h and complete bacterial elimination after 24 h. With 0.03 mg/L meropenem, bacteria were initially killed, although after 24 h, bacterial re-growth occurred up to the level of non-exposed bacterial growth after 24 h. However, this finding was not associated with changes in susceptibility to meropenem. Concentrations of ≤0.015 mg/L meropenem had no effect on in vitro bacterial growth. For *K. pneumoniae* KPC, initial killing by meropenem was observed at concentrations ≥0.5 mg/L during the first 2 h exposure, but bacterial re-growth was observed at all concentrations up to the level of non-exposed bacteria after 24 h. 

The mode of action of tigecycline was observed to be bacteriostatic and the antibiotic was equally effective against *K. pneumoniae* ESBL and *K. pneumoniae* KPC, indicating that the 2-fold difference in MIC values to tigecycline for the two strains represents essential agreement in susceptibility to this antibiotic. Inhibition of bacterial growth after 24 h of exposure was only observed above 2 mg/L tigecycline. Concentrations below 2 mg/L resulted in growth inhibition during 4 h of exposure, but bacterial re-growth occurred after 24 h, which was never associated with changes in bacterial susceptibility to tigecycline. Concentrations ≤0.25 mg/L had no effect.

### 3.4. Characterization of Pneumonia–Septicemia Model

**Figure 2:**
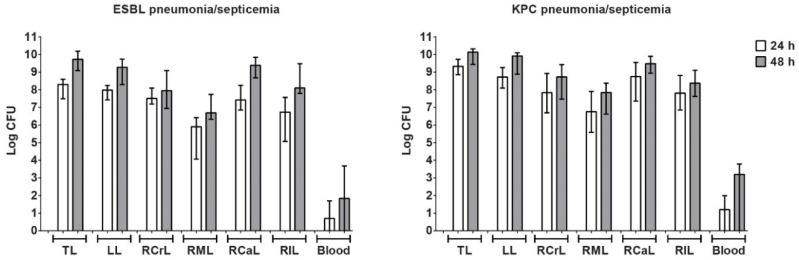
Bacterial load of *K. pneumoniae* in lungs and blood of rats with *K. pneumoniae* pneumonia–septicemia in the early phase of infection. Bacterial load of *K. pneumoniae* was determined for total lungs and individual lung lobes and blood (per mL) of rats with *K. pneumoniae* ESBL pneumonia–septicemia and rats with *K. pneumoniae* KPC pneumonia–septicemia at 24 h and 48 h after initiation of infection. TL, total lungs; LL, left lobe; RCrL, right cranial lobe; RML, right middle lobe; RCaL, right caudal lobe; RIL, right intermediate lobules. Groups of 6 rats per time point. Bacterial load is expressed as log CFU (median ± range).

**Figure A1:**
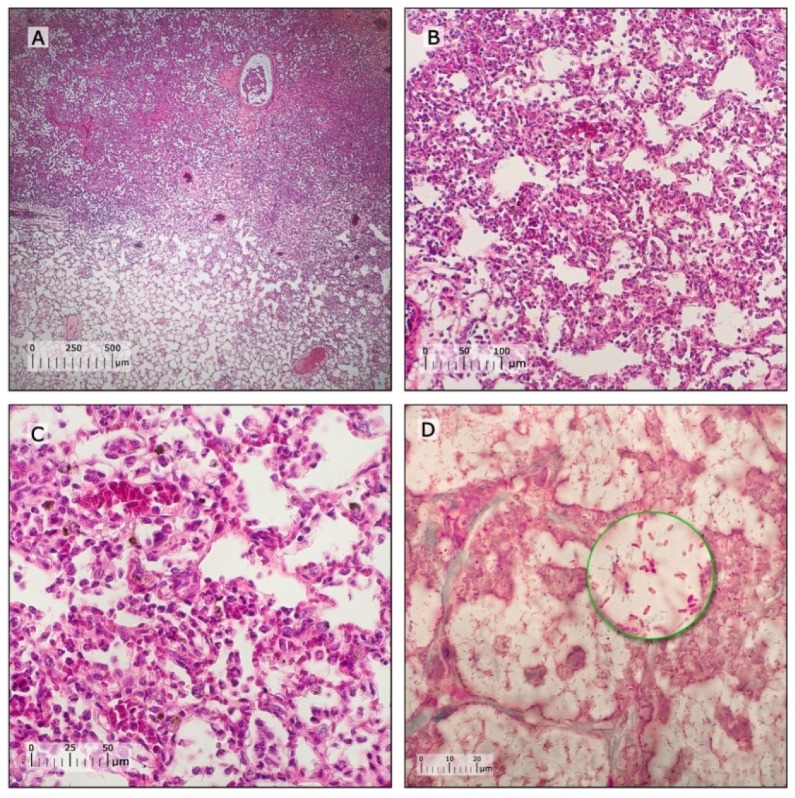
Representative lung histopathology in lung tissue from rats with *K. pneumoniae* KPC pneumonia–septicemia at 24 h after initiation of infection. (**A**–**C**) Sections stained with hematoxylin-eosin at different magnifications. (**D**) Section stained with Gram stain. An outer hemorrhagic zone of the lesion was observed (in areas next to uninfected tissue), in which the alveoli were filled with mucoid exudates dominated by edema fluid and a light cellular infiltrate. More extensive areas of pneumonia were observed towards the center of the lesion (i.e., in the zone of early consolidation). In these areas, alveoli were packed with predominantly polymorphonuclear leukocytes and alveolar walls were necrotic. Numerous encapsulated Gram-negative bacilli were freely present in the exudates within the hemorrhagic zone of the lesions.

Rats were inoculated with *K. pneumoniae* ESBL or *K. pneumoniae* KPC and developed acute bilateral pneumonia with septicemia. Bacterial numbers increased rapidly in all lung lobes, with the relatively large lobes containing the most bacteria (Figure 2). As a consequence of a higher inoculum used to induce KPC pneumonia–septicemia, higher counts of *K. pneumoniae* were found at 24 h in the lungs of rats with KPC pneumonia–septicemia compared to rats with ESBL pneumonia–septicemia. By 48 h, differences in *K. pneumoniae* counts in the lungs in both models were minor. After 24 h, bacteremia was present in 4/6 (66%) rats with ESBL pneumonia–septicemia and in 4/6 (66%) rats with KPC pneumonia–septicemia. Changes in liver and kidney function in terms of ALAT, ASAT, creatinine, and BUN were not found. Histopathological examination of the infected lungs at 24 h after initiation of infection revealed distinctive alveolar pathology, as shown for KPC pneumonia–septicemia (Figure A1 in Appendix A). Similar histopathological characteristics were observed in rats with ESBL pneumonia–septicemia. 

**Figure 3:**
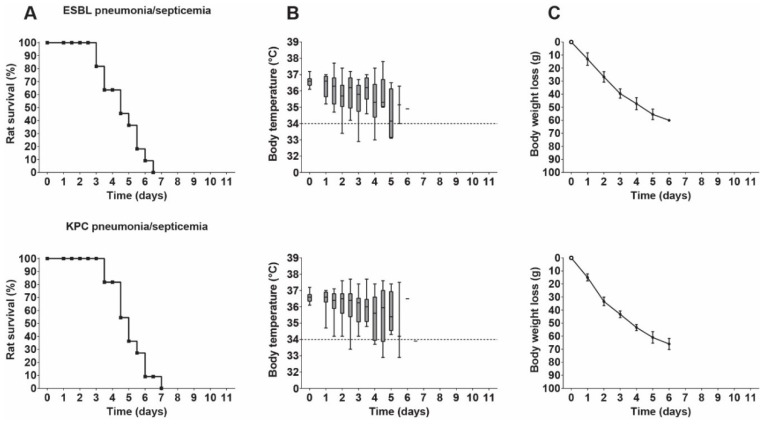
Disease progression in rats with *K. pneumoniae* pneumonia–septicemia. Groups of 11 rats were used for each experiment. (**A**) Rat survival (Kaplan–Meier curves) of rats reaching humane endpoints. (**B**) Box plots of pooled body temperatures showing the range of the data; the dashed line at 34 °C represents the humane endpoint. (**C**) Body weight loss from the onset of the infection (mean ± SD).

The disease progression over time in terms of rat survival rate (Figure 3A) was similar for either infecting *K. pneumoniae* strain (*p* = 0.4662). In both cases, the body temperatures (Figure 3B) initially showed some fluctuation, but decreased over time to below the lower limit (36.1 °C) of normal values. Rat body weight decreased over time (Figure 3C). A control experiment revealed that the technique of intubation and inoculation itself did not influence body temperature or body weight.

### 3.5. Therapeutic Efficacy of Meropenem in Rats with ESBL Pneumonia–Septicemia

**Figure 4:**
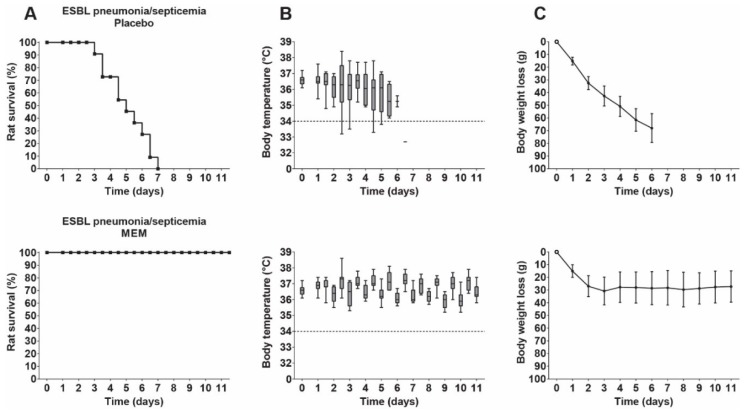
Therapeutic efficacy of meropenem (MEM) in rats with ESBL pneumonia–septicemia. Groups of 11 rats were treated 12-hourly for 10 days with placebo (physiological saline) or 25 mg/kg/day meropenem, starting at 24 h after initiation of infection. (**A**) Rat survival (Kaplan–Meier curves) of rats reaching humane endpoints. (**B**) Box plots of pooled body temperatures showing the range of the data; the dashed line at 34 °C represents the humane endpoint. (**C**) Body weight loss (mean ± SD).

Treatment was started at 24 h after infection, when bacterial numbers in the lung had increased 100-fold and most rats had developed early bacteremia. Increasing doses of meropenem were investigated, ranging from 6.25 to 25 mg/kg/day administered 12-hourly until a MED of meropenem was reached, at which all rats survived. Meropenem showed a dose-dependent therapeutic activity and effected survival of all rats at 25 mg/kg/day—the MED (Figure 4). Rat body temperatures initially showed some fluctuation but stabilized around normal values after 3 days, from which time onward normal circadian rhythm was shown. Body weight values initially decreased but remained stable after 3 days. The lungs of the surviving rats sacrificed at termination of the experiment were sterile. 

### 3.6. Pharmacokinetics of Meropenem in Rats with ESBL Pneumonia–Septicemia

**Figure 5:**
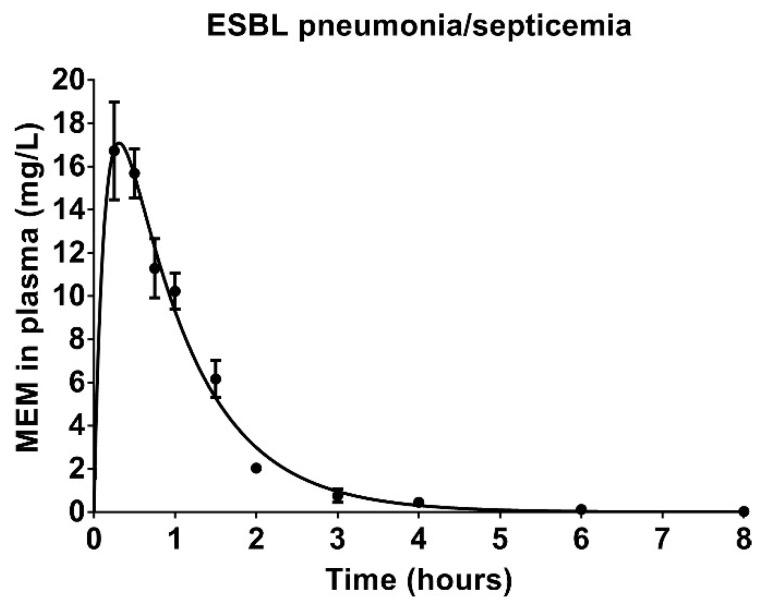
Plasma concentrations of total (bound and unbound) meropenem (MEM) in rats with ESBL pneumonia–septicemia. Rats were treated 12-hourly with 3 consecutive doses of 25 mg/kg/day meropenem, starting at 24 h after initiation of infection. The concentration–time curve predicted by a one-compartment model of extravascular administration was imposed over the observed concentrations at each time point in 3 rats (mean ± SEM).

Meropenem plasma concentrations were determined to investigate the steady-state pharmacokinetic profile of meropenem at MED in rats with ESBL pneumonia–septicemia. The plasma concentration–time curve indicated a one-compartment deposition (Figure 5). The corresponding estimated pharmacokinetic parameters are displayed in Table 2. The *ƒ*T > MIC for *K. pneumoniae* ESBL was 5.18 h, which was 43.17% of the twice-daily dosing interval. For *K. pneumoniae* KPC, the *ƒ*C_max_ (13.37 mg/L) did not reach the respective meropenem MIC (16 mg/L), resulting in a *ƒ*T > MIC of 0 h.

### 3.7. Therapeutic Efficacy of Meropenem and Tigecycline in Rats with KPC Pneumonia–Septicemia

**Figure 6:**
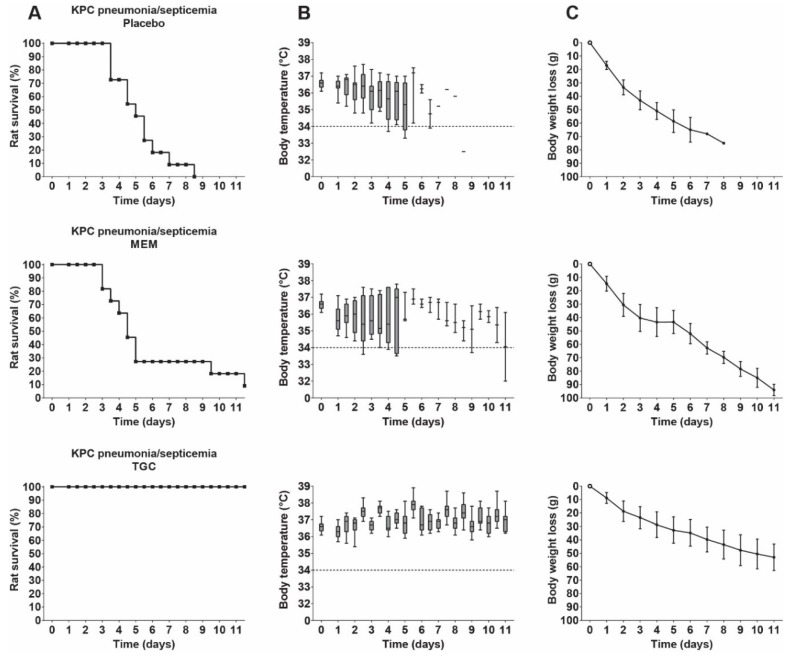
Therapeutic efficacy of meropenem (MEM) or tigecycline (TGC) in rats with KPC pneumonia–septicemia. Groups of 11 rats were treated 12-hourly for 10 days with placebo (physiological saline), 25 mg/kg/day meropenem, or 25 mg/kg/day tigecycline, starting at 24 h after initiation of infection. (**A**) Rat survival (Kaplan–Meier curves) of rats reaching humane endpoints. (**B**) Box plots of pooled body temperatures showing the range of the data; the dashed line at 34 °C represents the humane endpoint. (**C**) Body weight loss (mean ± SD).

Rats with KPC pneumonia–septicemia were treated with 25 mg/kg/day meropenem 12-hourly for 10 days, which correlates to a *ƒ*T > MIC of 0 h. This treatment resulted in survival of only 1/12 rats with KPC pneumonia–septicemia (Figure 6), which was not significantly different compared to the placebo-treated rats (*p* = 0.5728). The failure of meropenem treatment was also reflected in the body temperatures and the body weight values.

Next, the therapeutic response to tigecycline treatment of 25 mg/kg/day 12-hourly for 10 days was investigated in rats with KPC pneumonia–septicemia. This dosage was the MED of tigecycline found in a previous unilateral pneumonia–septicemia rat model caused by an identical *K. pneumoniae* ESBL strain [11]. The 25 mg/kg/day tigecycline dosage resulted in survival of all rats with bilateral KPC pneumonia–septicemia. Body temperatures remained stable, but body weights decreased slowly over time (Figure 6). Low bacterial numbers were present in the lungs of the surviving rats, with a median of 2.6 × 10^3^ *K. pneumoniae* and an upper limit of 1.9 × 10^4^ *K. pneumoniae*. None of the isolated bacteria showed a change in susceptibility to tigecycline.

## 4. Discussion

Antimicrobial resistance continues to spread worldwide and is often encountered in clinically relevant organisms such as *K. pneumoniae*. This bacterial species is becoming increasingly resistant to currently available antibiotics [3,34], with many isolates only susceptible to a limited number of “last resort” antibiotics, such as colistin and tigecycline. However, much debate still exists on the clinical efficacy of tigecycline in relation to the dose administered. Freire et al. in 2010 showed a lower therapeutic response for tigecycline than imipenem (using a dosing regimen of 100 mg initially followed by 50 mg every 12 h in a group of ventilated patients), a study often referenced as showing the limited efficacy of tigecycline in this situation [35]. Additionally, the administration of tigecycline treatment has been associated with an increased death rate [5], and a number of case reports have indicated serious complications in patients as a consequence of tigecycline treatment [36,37,38,39,40]. On the other hand, a meta-analysis by Falagas et al. in 2014 concluded that high-dose tigecycline regimens may be effective for the treatment of severe bacterial infections [9], and a meta-analysis by Gong et al. concluded that high-dose tigecycline regimens did not elevate the risk of toxic side effects [41]. Further, pharmacokinetic and pharmacodynamic studies have indicated that higher dosages of tigecycline can be administered to humans, resulting in improved therapeutic efficacy [6,42,43]. This has been corroborated by more recent studies, which have shown that administration of high-dose tigecycline regimens are tolerated and result in improved therapeutic efficacy in infected patients compared to conventional tigecycline dosing [43,44,45,46,47]. Cunha et al. reported clinical efficacy using once-daily high-dose tigecycline monotherapy in the treatment of multidrug-resistant Gram-negative bacilli and published recommendations for high-dose regimens [7,42]. These studies are also supported by the in vitro modeling studies of Tsala et al., suggesting that higher doses of tigecycline are required to achieve therapeutic efficacy [48], and by a Monte Carlo simulation analysis of standard and high-dose tigecycline against carbapenemase-producing *K. pneumoniae*, showing that the cumulative fractional response rate of >90% was only achievable with the high-dose tigecycline regimen [49]. Taken together, these studies suggest that current clinical guidelines on the antibiotic prescription of tigecycline may need to be revised upwards in order to reach a clinically effective dose. In this respect, EUCAST has recently published the new Guidance Document on Tigecycline Dosing by the EUCAST, recommending the use of high-dosage tigecycline treatment in seriously ill patients infected with multidrug-resistant bacteria [8].

Complicating this debate is the fact that tigecycline is frequently applied in the treatment of infections caused by multidrug-resistant bacteria as part of combination therapy rather than monotherapy [10]. Based on these limitations and on accompanying discussions relating to the efficacy of tigecycline administration, a study was established into the efficacy of high-dose tigecycline monotherapy using a newly established rat model of fatal acute pneumonia–septicemia caused by ESBL- or KPC-positive *K. pneumoniae* with meropenem as a comparator drug. This model is representative of a particularly severe multidrug-resistant infection in human patients [50,51,52] and allows for research comparing the therapeutic efficacy of individual antibiotics at similar conditions of severity, duration of infection, and host defense, which is not possible with most comparative clinical efficacy studies on tigecycline treatment [9,10].

Treatment with tigecycline at 25 mg/kg/day 12-hourly for 10 days resulted in 100% survival of rats with KPC pneumonia–septicemia and normalization of body temperature. In our previous study using the *K. pneumoniae* ESBL unilateral pneumonia–septicemia model, this tigecycline dosage was the successful MED, generating 100% survival of rats and normalization of body temperature. In the same study, the efficacy of tigecycline was found to be dose-dependent and was correlated to the ratio of the area under the plasma concentration–time curve (AUC) to the MIC (AUC/MIC). The AUC during a 24-h period (AUC_0–24h_) for this tigecycline dosage in rats with was similar to the AUC_0–24h_ of high-dose tigecycline treatments in human patients (a loading dose of 300 mg tigecycline followed by 150 mg tigecycline every 12 h) [53]. For pneumonia–septicemia caused by *K. pneumoniae* ESBL with a tigecycline MIC of 1 mg/L, the AUC/MIC was 34.29 mg·h/L at a tigecycline dosage of 25 mg/kg/day, which was the MED of tigecycline for survival of all rats. As the tigecycline MIC of *K. pneumoniae* KPC is 2 mg/L, the extrapolated AUC/MIC ratio for KPC pneumonia–septicemia at a tigecycline dosage of 25 mg/kg/day is calculated to be 17.15 mg·h/L. Despite this MED having a lower AUC/MIC ratio in rats with bilateral KPC pneumonia–septicemia, the tigecycline treatment at 25 mg/kg/day 12-hourly for 10 days remained successful. Unlike the meropenem treatment, this treatment did not result in normalization of body weight, which might be explained by either the persistence of low bacterial numbers in the lungs of the rats at the end of treatment or by potential gastro-intestinal side effects of tigecycline.

Treatment with meropenem in rats with ESBL pneumonia–septicemia showed a clear dose–response relationship. At the MED of 25 mg/kg/day, meropenem resulted in 100% rat survival, stabilization of body weight, body temperature returning to the circadian rhythm, and effected bacterial elimination from the lung tissue at the end of treatment. Meropenem concentrations in rat plasma remained above the MIC of *K. pneumoniae* ESBL for >5 h, achieving the bactericidal target of carbapenem regimens in human patients, which is a ƒT > MIC of approximately 40% of the dosing interval [54]. In terms of total dosage, meropenem at 25 mg/kg/day 12-hourly for 10 days in rats is similar to the current clinical treatment of ESBL pneumonia–septicemia, although in patients, an 8-hourly meropenem schedule is more common [55].

The MED of meropenem in rats with ESBL pneumonia–septicemia was not successful in rats with KPC pneumonia–septicemia, which is in line with the difference in susceptibility of *K. pneumoniae* ESBL and *K. pneumoniae* KPC. This difference in susceptibility resulted in different *ƒ*T > MIC values for the two strains, with unbound meropenem concentrations in rat plasma remaining above the MIC of *K. pneumoniae* ESBL for >5 h, whereas the ƒC_max_ (<14 mg/L) did not reach the meropenem MIC of *K. pneumoniae* KPC (16 mg/L), resulting in a *ƒ*T > MIC of 0 h. The respective therapeutic success or failure of this meropenem dose in both infection models can be explained as a consequence of the time-dependent bactericidal activity of meropenem, which was shown in the in vitro TKK data in the present study, as well as in other in vivo pharmacokinetic and pharmacodynamic studies [56].

The pneumonia–septicemia model established in the present study is representative of a particularly severe human infection with *K. pneumoniae* bacteria, resulting in rapid mortality when left untreated [50,51,52]. Further, although the tigecycline MIC of *K. pneumoniae* ESBL (1 mg/L) and *K. pneumoniae* KPC (2 mg/L) are considered resistant, these MICs remain within the wild-type distribution of tigecycline MICs for *K. pneumoniae* [26,28]. In view of these factors, the successful treatment of this animal model necessitated the use of a high-dose tigecycline regimen, as the AUC of the tigecycline monotherapy MED administered to rats with ESBL pneumonia–septicemia exceeds the AUC of the tigecycline dosage that is conventionally administered to infected patients (50 mg tigecycline every 12 h) [57], demonstrating the effectiveness of high-dosage tigecycline regimens in the treatment of severe multidrug-resistant bacterial infections. The scope of the current research was limited to investigating the efficacy of a high-dosage tigecycline regimen in a model of pneumonia–septicemia caused by two isogenic, multidrug-resistant *K. pneumoniae* strains. Future research can improve on the generalizability of these results by investigating high-dosage tigecycline regimens in the treatment of infection models caused by other *K. pneumoniae* strains or different Gram-negative bacterial species. Another interesting avenue for further research with the current pneumonia–septicemia model would be the investigation of high-dose meropenem regimens [58] or high-dose meropenem and high-dose tigecycline combination regimens [59].

## 5. Conclusions

The data obtained in the current study provide evidence that high-dosage tigecycline monotherapy is effective in a rat model representative of human pneumonia–septicemia caused by multidrug-resistant *K. pneumoniae*. Based on the therapeutic tigecycline dose observed in this publication, the limited susceptibility to tigecycline of both strains, and current clinical guidelines, the in vivo data reported in the present study further supports recent literature on the applicability of high-dosage tigecycline as a treatment of “last resort” in patients for severe multidrug-resistant *K. pneumoniae* infections. The present work involves a clinically relevant animal model for multidrug-resistant *K. pneumoniae* infections that facilitates the future investigation of antibiotics and novel therapeutic approaches to pneumonia–septicemia.

## Figures and Tables

**Table 1:** Minimum inhibitory concentration (MIC) values of clinically relevant antibiotics for *K. pneumoniae* strains.

Strain		ATCC 43816	EMC 2003	EMC 2014	ATCC 13883	ATCC 700603	ATCC BAA1705
Phenotype		WT Parent	ESBL	KPC	WT	ESBL	KPC
**MIC (mg/L)**	**CAZ**	0.5 ^S^	256 ^R^	256 ^R^	0.5 ^S^	64 ^R^	128 ^R^
	**MEM**	0.063 ^S^	0.063 ^S^	16 ^R^	0.063 ^S^	0.063 ^S^	32 ^R^
	**AMK**	2 ^S^	2 ^S^	2 ^S^	1 ^S^	1 ^S^	32 ^R^
	**TOB**	0.5 ^S^	≥128 ^R^	≥128 ^R^	0.5 ^S^	8 ^R^	32 ^R^
	**CIP**	0.031 ^S^	0.125 ^S^	0.063 ^S^	0.125 ^S^	0.5 ^I^	≥64 ^R^
	**NOR**	0.125 ^S^	0.25 ^S^	0.25 ^S^	0.25 ^S^	2 ^R^	512 ^R^
	**SXT**	0.5 ^S^	1 ^S^	2 ^S^	0.5 ^S^	4 ^I^	≥128 ^R^
	**CST**	0.5 ^S^	1 ^S^	0.5 ^S^	2 ^S^	1 ^S^	0.5 ^S^
	**TGC**	1 ^R^	1 ^R^	2 ^R^	1 ^R^	16 ^R^	4 ^R^

MIC assays were performed in triplicate; median values are displayed. MIC values were interpreted as susceptible (^S^), intermediate (^I^), or resistant (^R^) according to EUCAST 2020 guidelines. ATCC, American Type Culture Collection; EMC, Erasmus University Medical Center Rotterdam; CAZ, ceftazidime; MEM, meropenem; AMK, amikacin; TOB, tobramycin; CIP, ciprofloxacin; NOR, norfloxacin; SXT, trimethoprim/sulfamethoxazole expressed as the trimethoprim concentration; CST, colistin; TGC, tigecycline; WT, wildtype; ESBL, extended-spectrum β-lactamase; KPC, *Klebsiella pneumoniae* carbapenemase.

**Table 2:** Pharmacokinetic parameters of meropenem in rats with ESBL pneumonia–septicemia.

Pharmacokinetic Parameter	V/F	CL/F	t_1/2_	*ƒ*Cmax	*ƒ*T > MIC			
					0.063 mg/L		16 mg/L	
	L/kg	L/kg/h	h	mg/L	h	%	h	%
**Estimate**	1.03	1.17	0.61	13.37	5.18	43.17	0.00	0.00
**SEM**	0.07	0.06	0.03	1.43	0.24	2.00	0.00	0.00

Plasma concentrations of meropenem in rats with ESBL pneumonia–septicemia treated 12-hourly with 3 consecutive doses of 25 mg/kg meropenem, starting at 24 h after initiation of infection. The *ƒ*T > MIC was calculated based on unbound meropenem concentrations using the MIC of *K. pneumoniae* ESBL (0.063 mg/L) and the MIC of *K. pneumoniae* KPC (16 mg/L) and is shown in hours (h) as well as percentage of the 12-h dosing interval (%). V, volume of distribution; F, bioavailability; CL, clearance; t_1/2_, elimination half-life time; *ƒ*Cmax, highest unbound meropenem concentration reached in blood plasma; *ƒ*T > MIC, cumulative percentage of a 12-h period that the unbound fraction of drug exceeds the MIC.

## Supplementary Materials

The following are available online at https://www.mdpi.com/2079-6382/9/3/109/s1: Table S1: Genotypic characterization of *K. pneumoniae* strains—MLST data and PCR data. Table S2: Phenotypic characterization of *K. pneumoniae* strains—VITEK^®^ MIC values of clinically relevant antibiotics.
