# Supplementary material for: Successful High-Dosage Monotherapy of Tigecycline in a Multidrug-Resistant Klebsiella pneumoniae Pneumonia–Septicemia Model in Rats
Source: Antibiotics (Basel). 2020 Mar 3;9(3):109. doi: 10.3390/antibiotics9030109 (PMC7148456; doi:10.3390/antibiotics9030109)
Supplement: Supplementary file 1 [file antibiotics-09-00109-s001.zip › Supplementary Files.docx]

**Supplementary Table S1.** Genotypic characterization of *K. pneumoniae* strains – MLST data and PCR data

| **Strain** | | ATCC 43816™ | EMC  2003 | EMC  2014 | ATCC 13883™ | ATCC 700603™ | ATCC BAA1705™ |
| --- | --- | --- | --- | --- | --- | --- | --- |
| **Antibiotic profile** | | WT Parent | ESBL | KPC | WT | ESBL | KPC |
| **Sequence type (ST)** | | 493 | 493 | 493 | 3 | 489 | 258 |
| **CTX-M** | **1** | negative | negative | negative | negative | negative | negative |
|  | **2** | negative | negative | negative | negative | negative | negative |
|  | **8** | negative | negative | negative | negative | negative | negative |
|  | **9** | negative | negative | negative | negative | negative | negative |
|  | **25** | negative | negative | negative | negative | negative | negative |
| **TEM** | | negative | negative | **positive** | negative | negative | **positive** |
| **SHV** | | **positive** | **positive** | **positive** | **positive** | **positive** | **positive** |
| **OXA** | **1** | negative | negative | negative | negative | negative | negative |
|  | **48** | negative | negative | negative | negative | negative | negative |
| **KPC** | | negative | negative | **positive** | negative | negative | **positive** |
| **NDM-1** | | negative | negative | negative | negative | negative | negative |

STs were determined by HiMLST for K. pneumoniae WT, *K. pneumoniae* ESBL EMC2003,
*K. pneumoniae* KPC EMC2014 and *K. pneumoniae* reference strains. Both strains contained an SHV-5 gene, and the *K. pneumoniae* KPC strain had gained an additional KPC gene. Both
*K. pneumoniae* strains belonged to the same sequence type (ST 493) compared to the WT parent strain, but were genetically different compared to the three ATCC reference strains, which each belonged to a different sequence type (ATCC 13883, ST 3; ATCC 700603, ST 489; ATCC BAA1705, ST258) indicating the genetic diversity among these strains.

**Supplementary Table S2.** Phenotypic characterization of *K. pneumoniae* strains - VITEK® MIC values of clinically relevant antibiotics

| **Strain** | ATCC  43816™ | EMC  2003 | EMC  2014 | ATCC  13883™ | ATCC  700603™ | ATCC  BAA1705™ |
| --- | --- | --- | --- | --- | --- | --- |
| **Antibiotic profile** | WT Parent | ESBL | KPC | WT | ESBL | KPC |
| Ampicillin | **16** | **≥32** | **≥32** | **≥32** | **≥32** | **≥32** |
| Amoxicillin/ Clavulanic Acid | ≤2 | 4 | **≥32** | ≤2 | **16** | **≥32** |
| Piperacillin/ Tazobactam | ≤4 | ≤4 | **≥128** | ≤2 | **32** | **≥128** |
| Cefuroxime | 2 | **16** | **≥64** | 4 | **≥64** | **≥64** |
| Cefuroxime Axetil | 2 | **16** | **≥64** | 4 | **≥64** | **≥64** |
| Cefoxitin | ≤4 | ≤4 | ≤4 | ≤4 | **≥64** | **≥64** |
| Cefotaxime | ≤1 | *2* | **8** | ≤1 | **8** | **8** |
| Ceftazidime | ≤1 | **≥64** | **≥64** | ≤1 | **32** | **16** |
| Cefepime | ≤1 | ≤1 | *2* | ≤1 | ≤1 | *2* |
| Imipenem | ≤0.25 | ≤0.25 | **≥16** | 0.5 | ≤0.25 | **≥16** |
| Meropenem | ≤0.25 | ≤0.25 | **≥16** | ≤0.25 | ≤0.25 | **≥16** |
| Gentamicin | ≤1 | **≥16** | **≥16** | ≤1 | **≥16** | **4** |
| Tobramycin | ≤1 | **≥16** | **≥16** | ≤1 | **8** | **≥16** |
| Ciprofloxacin | ≤0.25 | ≤0.25 | ≤0.25 | ≤0.25 | *0.5* | **≥4** |
| Norfloxacin | ≤0.5 | ≤0.5 | ≤0.5 | ≤0.5 | **2** | **≥16** |
| Trimethoprim | ≤0.5 | ≤0.5 | ≤0.5 | ≤0.5 | 2 | **≥16** |
| Trimethoprim/ sulfamethoxazole | ≤1 | ≤1 | ≤1 | ≤1 | ≤1 | **≥16** |
| Colistin | ≤0.5 | ≤0.5 | ≤0.5 | ≤0.5 | ≤0.5 | ≤0.5 |

MICs (mg/L) were determined using the automated VITEK®2 antimicrobial identification system for *K. pneumoniae* WT*, K. pneumoniae* ESBL EMC 2003, *K. pneumoniae* KPC EMC 2014 and
*K. pneumoniae* reference strains. Trimethoprim/sulfamethoxazole was expressed as the trimethoprim concentration. Interpretation of antimicrobial susceptibility was defined by the VITEK® antimicrobial identification system as susceptible, intermediate (italic), or resistant (bold) based on EUCAST 2014 guidelines. *K. pneumoniae* ESBL EMC2003 and *K. pneumoniae* KPC EMC2014 had few major differences in susceptibility profile compared to ATCC reference strains.
